# Ethical issues relating to the banking of umbilical cord blood in Mexico

**DOI:** 10.1186/1472-6939-10-12

**Published:** 2009-08-14

**Authors:** V Moises Serrano-Delgado, Barbara Novello-Garza, Edith Valdez-Martinez

**Affiliations:** 1 Obstetrics and Gynaecology Hospital with Family Medicine # 13, Mexican Institute of Social Security, Mexico City, Mexico; 2 Dirección de prestaciones médicas, Mexican Institute of Social Security, Mexico City, Mexico; 3 Health Research Council, Mexican Institute of Social Security, Mexico City, Mexico

## Abstract

**Background:**

Umbilical cord banks are a central component, as umbilical cord tissue providers, in both medical treatment and scientific research with stem cells. But, whereas the creation of umbilical cord banks is seen as successful practice, it is perceived as a risky style of play by others. This article examines and discusses the ethical, medical and legal considerations that arise from the operation of umbilical cord banks in Mexico.

**Discussion:**

A number of experts have stated that the use of umbilical cord goes beyond the mere utilization of human tissues for the purpose of treatment. This tissue is also used in research studies: genetic studies, studies to evaluate the effectiveness of new antibiotics, studies to identify new proteins, etc. Meanwhile, others claim that the law and other norms for the functioning of cord banks are not consistent and are poorly defined. Some of these critics point out that the confidentiality of donor information is handled differently in different places. The fact that private cord banks offer their services as "biological insurance" in order to obtain informed consent by promising the parents that the tissue that will be stored insures the health of their child in the future raises the issue of whether the consent is freely given or given under coercion. Another consideration that must be made in relation to privately owned cord banks has to do with the ownership of the stored umbilical cord.

**Summary:**

Conflicts between moral principles and economic interests (non-moral principles) cause dilemmas in the clinical practice of umbilical cord blood storage and use especially in privately owned banks. This article presents a reflection and some of the guidelines that must be followed by umbilical cord banks in order to deal with these conflicts. This reflection is based on the fundamental notions of ethics and public health and seeks to be a contribution towards the improvement of umbilical cord banks' performance.

## Background

The extraordinary scientific and technological advance of contemporary medicine constantly leads toward the introduction of new treatments, as well as treatments for illnesses that were previously untreatable; one example of this is the discovery and introduction into clinical medicine of the use of umbilical cord blood for the acquisition and transplantation of stem cells to treat bone marrow disorders, both genetic and acquired [1-3]. Similarly, the hypothesis that these cells have enormous therapeutic possibilities in different illnesses is being actively researched. These illnesses affect the following body systems: cardiovascular, neurological, gastrointestinal, bone and joint, endocrine and skin. The acquisition of stem cells from umbilical cord blood for the treatment of these illnesses brings with it the obligation to create and operate umbilical cord banks (UCB) in a manner that is congruent with the ethics of clinical health care and public health [4].

When they are classified by the source of their funding, UCBs may be publicly funded or funded for profit. Publicly funded banks, as created by the government, promote the altruistic donation; and, even though these banks store umbilical cord tissue in order to use it to benefit any person who may need it, they also store samples of the donated tissue for autologous transplant in certain selected cases. In contrast, for profit banks, created with private capital, require that the cost of the service be paid by the donors, and the donated tissue is meant for autologous use and not for transplants to others, although occasionally this may happen [1,3].

These umbilical cord banks first appeared in the world in the 1980s. At present there are approximately 107 of them worldwide and the majority is publicly owned. Official data on these banks reveals that by the year 2007 the number of samples stored was 406,000; the number of allogenic transplants was 2,743; 79% of the cord blood units were used for single or double transplantation [5]. These data also reveal that there is a minimal autologous utilization of the tissue stored, which is estimated to be 1 in 20,000 samples stored during the first 20 years of life of the donor [6]. These data support the projections of Ballen who estimates that the probability of using one's own cord blood varies between 1:2500 (0.04%) and 1:200,000 (0.0005%) in the first 20 years of life [7,8]. It is interesting to note that in almost every case in the United States where a family was caught by surprise by a deadly disease and a transplant was needed, a cord blood match was found at a public blood bank, such as those at Georgetown University Medical Center, the New York Blood Center, UCLA or Duke University [9]. In the United Kingdom, a bank sized of 50,000 could ensure at least one donor unit available for 80–98% of the population [10].

In Mexico there are three public banks: "CordMx" which belongs to the National Center for Blood Transfusion of the Federal Department of Health, the Bank of the University Hospital of Nuevo Leon, and the Central Blood Bank of the "La Raza" National Medical Centre which is part of the Mexican Institute of Social Security [11]. CordMx is the only Latin American bank to be registered with Netcord, an international foundation which affiliates umbilical cord blood banks [12]. CordMx belongs to the World Marrow Donor Association (WMDA). On the basis of the percentage of cord blood units (CB) provided for transplantation in relation to the number of CB units present, in 2007, CordMx was first in the worldwide list of banks. The utilization rate was 3.79%. Furthermore, as regards to the percentage of CB units exported, this public bank was thirteenth in that list [5].

Mexico is in fourth place worldwide in the number of private banks it has; it is estimated that there are at least one dozen of them [13]. Private banks actually cover more geographical areas than public banks. Since more than half of the population of the country cannot afford the services of private banks, and Mexico has not yet enacted specific legislation to regulate them, there are issues about the ethics of their operation. The table 1 summarizes some of the differences in the operation of Umbilical Cord Blood Banks in Mexico.

**Table 1:** Some of the differences in the operation of Umbilical Cord Blood Banks in Mexico

Private banks	Public banks
Assure compatibility in 100% of cases.	Compatibility reaches up to 90%.
The possibility of use by other members of the family of the donor is mentioned. This is an affirmation which is part of their marketing strategy.	A bioarchive of Histoleukocytic antigen is available to increase the possibility of compatibility.
The availability of the unit is immediate; however samples are regularly stored in foreign banks.	Availability is not immediate. A bioarchive assures better compatibility.
Storage costs are paid by the donor. On the average an initial payment of 800 USD is required as well as annual payments of 90 USD. Financial plans for 5, 10, 15 and even 20 years are available.	No cost to the donor; all costs are paid by the State. They promote solidarity through the altruistic donation of umbilical cords. Any person may obtain a sample for medical treatment without the need to pay.
No evidence that NOM-003-SSA2-1993 or quality certificate ISO 9001–2000 are being complied with; and no evidence of affiliation to international groups that would certify the adherence to quality standards.	Operation complies with international quality controls (World Association of Bone Marrow Transplants, International Foundation Netcord, American Association of Blood Banks AABB, Norma Oficial Mexicana NOM-003-SSA2-1993, Quality certifications ISO 9001–2000.
Despite the absence of certified quality control for the storage and transport of the samples, all umbilical cord samples are stored.	Strict quality controls for the selection of donors, the acquisition, storage, transport and cellular viability. Only samples that meet strict standards of quality and cellular viability are stored.
The acquisition of samples is usually completed by a third party; thereby the responsibility to complete this task in a prescribed manner is diluted. This also creates the possibility of clandestine payments to those who acquire the samples; this would bias the sample acquisition process in favor of the private banks.	Acquisition is completed by trained personnel.

Up to June 2009, the umbilical cord blood bank at the "La Raza" National Medical Centre reported 500 tissue samples in storage, and to have performed 37 transplants; this number represents an utilization rate of 7.4% of the tissue stored. In order to guarantee the quality of the stored tissue, this UCB does a follow up of all tissues and donors during a three to six month period [Guerra-Marquez A. Unpublished observations].

Research has shown that the HLA (Human Leukocyte Antigen) compatibility of a transplant is smaller in allogenic transplants than in autologous transplants; nevertheless, the storage capacity of public banks promotes availability and genetic diversity [7,10,14,15]. Table 2 summarizes some of the major therapeutic indications for the transplant of haematopoietic stem cells approved by the Ministry of Health in Mexico [16]. But, the American Academy of Pediatrics and American Society of Haematology extend the list of therapeutic indications to other disorders such as: malignancies, bone marrow failfure, hemoglobinopathies, inmunofeficiencies, and inborn errors of metabolism [15,17-20]. The Iberoamerican Council of Mar de La Plata [2005] issued a proposal recommending that the autologous use of the tissue be restricted and that the creation of private, for-profit banks be prohibited [21]. The Iberoamerican Council bases its recommendation on the following facts: (i) the tissue stored in private banks is seldom used; (ii) autologous use assures complete compatibility, but there is no scientific evidence that may justify the storage of tissue samples indefinitely; (iii) the obligation of governments to preferentially promote treatments of efficacy as proven scientifically; (iv) the importance of taking into consideration the results of a cost-benefit analysis.

**Table 2:** Indications for the transplant of haematopoietic stem cells

Allogenic	Autologous
**Congenital Diseases**	
Congenital combined immunodeficiency	*
Fanconi's medular aplasia	*
Talasemia major	*
Sickle cell	*
Blackfan-Diamond's erythroblastopenia	*
Kostmann's neutropenia	*
Juvenile Osteopetrosis	*
Thesaurismosis	*
Chronic Granulomatous Disease	*
**Acquired Neoplastic Diseases**	
Acute Leukemias	*
Chronic Myeloid Lukemia	*
Chronic Lymphocytic Leukemia	*
Non-Hodkin Lymphoma	*
Hodgkin Lymphoma	*
Multiple Myeloma	*
Histiocytosis	*
Myelodysplastic Syndrome	*
Solid Tumors	*
**Non-neoplastic acquired diseases**	
Severe Marrow Aplasia	*

* No indication

Table adapted from: Asociación Mexicana de Medicina Transfusional, A.C., Agrupación Mexicana para el Estudio de la Hematología, A.C. Guía para el uso clínico de la sangre. México: Secretaría de Salud; 2007.

The European Group on the Ethics of Science and New Technologies states that the donation of human tissues must be freely done, that it must be altruistic, and that the donor does not have to receive remuneration. For this group it is also important that the donation be based on the notion of solidarity, and the donor not be considered as an object that provides organs and tissues, but as a person. And the group is emphatic on the need to avoid the exploitation of the more disadvantaged social classes, the class more likely to donate when the donations are not altruistic [22]. Italy is the only country of the European Union which expressly prohibits the existence of privately owned, for-profit, BCUs because the allogenic use of stem cells from the umbilical cord is still under investigation [6].

The American Academy of Pediatrics through its Pediatric BCU Group discourages parents from using private storage facilities, despite the fact that they acknowledge the possibility of future autologous use, above all, in illnesses with genetically determined immune deficiency, and in some other acquired diseases of the bone marrow. They also believe that the parents are subjected to the undue influence, coercion and manipulation by private banks in order to obtain consent for the storage of blood at the moment of birth, taking advantage of the emotions that are displayed on such an occasion [15].

There are a number of ethical, legal and social problems lurking behind the operation of these banks. This article presents a meticulous review of the topics related to the UCB, and analyzes the ethical, legal, health policy, medical and scientific considerations that allow their operation in Mexico.

## Discussion

### Public policy and legal considerations

There is a close relation between law and public policy. In this article "public policy" refers to a set of normative guidelines which a governmental entity may demand to be followed. These public policies may prescribe that individuals and organizations behave or not behave in a certain way.

What has been discussed above signals the need for the definition of public policy and law to regulate all aspects of the functioning of UCBs, in particular: (1) what kind of installations and trained personnel must an entity have before it bills itself as a UCB, (2) what kind of standards must the operations of these installations and personnel meet, (3) who will certify that the standards are being met at any given time, (4) how will informed consent to store the tissue be obtained, (5) who will pay for the indefinite storage of the tissue, and (6) who and under what circumstances will that person have access to the tissue stored. But, this raises the following questions: What kinds of interests are involved in the operation of private banks? How can the State intervene to fill the fundamental health needs of a population? What is fair in the acquisition of samples? What is fair in accessing the samples stored? What kind of input toward policy definition should come from the existing public and private UCBS when the criteria that define good practice are defined?

Despite the fact that there are international entities that publish guidelines for the operation of UCBs, the majority of nations do not have a specific legal framework for the operation of these banks, nor do they have the health regulations that are in agreement with technological development [1,6]. In Mexico, the therapeutic use of human blood is regulated by: (**a**) the General Health Law (1984) that establishes the basis and modes for the provision of health care and for basic and applied health research. However, the law is general and does not define the manner in which the UCBs will be regulated. (**b**) The official Mexican norm (NOM-003-SSA2-1993) [23], which must be observed throughout the nation by the public and the private sectors, regulates the therapeutic uses of human blood and its components. However, it does not consider the utilization of stem cells derived from umbilical cord blood. The norm is limited to defining the manner and conditions under which the following should be carried out: the allogenic disposal and selection of the donor, the collection and safekeeping, handling, storage and control of all blood units and their components. (**c**) The document on the clinical use of blood that was published by the Health Ministry jointly with the Mexican Association of Transfusion Medicine, A.C., and the Mexican Group for the Study of Haematology, A.C., This document defines, in Chapter VIII, the guidelines to be followed in order to maintain standards of quality during the acquisition, storage and use of stem cells from the umbilical cord [16]. The document does not recommend autologous donations because of the probability of the appearance of cancer, congenital autoimmune deficiency or genetic diseases associated with this procedure as reported in the literature [16]. Nevertheless, it does not prohibit the storage of samples from umbilical cord in private banks. In this respect it must be remembered that "The fact that an act is morally acceptable does not mean that the law must permit it." [37].

The absence of these guidelines from Mexican legislation implies the need for a serious reflection on the moral responsibility of the legislative branches of government previous to the definition of public law. This reflection must consider, at least the six aspects of UCBs that must be regulated with particular emphasis on the following:

(a) The process of informed consent should be previous to the donation and storage of blood from the umbilical cord. International organizations recommend that the written permission for obtaining cord blood should be obtained long before onset of active labor, so that parents will be able to decide freely and responsibly [24,25]. It is interesting to note that the American Pediatrics Association and the American College of Obstetrics and Gynecology doubt the validity of informed consent obtained by private banks. They state that these banks coerce and manipulate parents in order to obtain their consent. The banks lead them to believe that the possible treatment of a future disease is in their hands, and they offer the service as some sort of "biological insurance." This is a situation that may generate guilt in the parents involved when they cannot buy their offspring a "hope for life" [12,26-28].

(b) The fact that ownership of the cord stored in private banks passes to the child (donor) once he/she has come of age [29]. This gives rise ethical concerns about what future use will be made of tissue that is not used; what is the reason that justifies a lifetime autologous storage.

(c) Confidentiality of donor information. In private banks this is not strictly kept [30]. For example, in Spain, the Health Ministry, which was initially opposed to the creation of private banks, allowed it upon being required to store the tissue of the first daughter of the Prince of Asturias, the first in line to inherit the Spanish crown. Information about the storage of this tissue was in the public domain [31-34], and as a consequence, the Spanish Government issued a decree (2006) stating that "the stem cells of private banks will be used publicly"; therefore, any ill person who may need a transplant and for whom compatible cells may be found in private banks will be able to use them [35,36].

All of this does not necessarily imply that Mexican law should prohibit the establishment and operation of private banks, but it does evidence the need for the existence of laws, norms and regulations to govern their operation and the operation of publicly funded banks. And for this, it is important to state that the authors of this article believe that there is a natural right to store blood from the umbilical cord and that any positive law about the storage and use of UCB must be based upon it; and through this positive law the legislative branches of government must to seek the good of order – the common good – of the population through which the particular good represented by the stored blood will be available to individuals. With this consideration there emerges the need consider the notion justice, which is nothing else but the willingness to give each what is due to him or her, that is, what corresponds to each [37].

### Ethics, morals and UCBs

The ethical reasoning process has as its objective to reach clarity, systematic order and precision in the arguments through which we make a decision about what is good for populations and individuals [37]. Therefore, ethical analysis must be systematic, and comply with the very demands that are defined by reason: (1) be attentive to all of the data available, (2) be intelligent and understand the data, (3) be rational and judge whether the understanding is correct or not, or probably correct or incorrect, (4) be responsible and decide on the basis of what has been correctly understood [38,39].

At present, ethical thinking is polarized between those who hold that there is a set of principles which is common to all human beings, independently of differences in religion, politics and culture; and those who hold that ethical principles are elements of a culture and different cultures have different principles. Those who belong to the first group assert that it is in judgments and decisions made on the bases of these common principles, and also based on the ethical traditions of medicine that the principles of beneficence, non-maleficence, autonomy and justice may be discovered [37,38]. Those that belong to the second group state that there are no universal principles in ethics, that is, there are no ethical principles which are valid for all peoples for all time [40]. These two opposite perspectives are important when reflecting upon the need for legislation to regulate the operation of UCBs. However, this paper is written from the position that there are certain ethical principles which are universal. These principles *prima facie *are used as the framework of reference for the analysis it presents. This article is written from a position that states that these principles constitute the basis for the solution of ethical dilemmas. And, when these principles are combined with the idea of public health as an element of the common good (and as long as they are not reduced to algorithms or rules from which all correct answers to moral problems may be derived) they can provide the ethical standard that allows for the evaluation of the operation of UCBs and the roles of everyone who is involved in their operation, including: the UCB themselves, and the donors, physicians/researchers as well as the communities where they operate.

### Beneficence

The ethical obligation to benefit the patient, while exposing him/her to the smallest possible risk, is the basis for the use of haematopoietic stem cells from the blood of the umbilical cord *in specific diseases *(Table 2). This principle is ignored or nor appropriately understood by private banks when, on the one hand, they offer a service based on false promises, such as that of "biological insurance," for which there is no scientific evidence; on the other hand, deceiving donors by assuring them that the tissue will remain in storage and available to be used at any moment of the life of their child. It is appropriate to underline that given the amount of tissue stored, it would be absolutely necessary to use allogenic donors if it were necessary to transplant an adult or if a patient (at any age) required of a second transplant [19,20]. As to the idea of cellular expansion (using *in vitro *techniques) it must be stated that this is something which is still being investigated [20,41]

### Non maleficence

The traditional moral obligation of Hippocratic medicine is *primum non nocere*, to first do no harm. In private banks, as opposed to public banks, it is not known if the criteria for quality control are followed and no harm is done. These banks have no rigorous control over such as: cellular viability, sample volume, method of acquisition, handling and transportation [11,42,43]. How then, could the quality of the tissue transplanted be guaranteed when the donor needs to use it?

Private banks should absorb their own quality control costs as any other commercial enterprise. The Government's job is to create health policies and regulations that guarantee the ethical functioning of both types of UCBs [44].

### Autonomy

When the State guarantees the right of persons to choose freely, it shows that the autonomy – self rule – of humans is acknowledged by it and respected. The basis for informed consent resides on the idea that competent persons are by nature autonomous and the best guardians of their own welfare. Public banks, in contrast to what happens in private banks, allow donors to exercise freely and responsibly the right to choose to store the tissue, when conflicts of interest are absent, due to the altruistic nature of the donation.

With this in mind, then why would parents choose to store blood from the umbilical cord of their child in a private bank instead of doing in it a public bank? A number of ethical question emerge immediately around this question: can you speak of respect for autonomy – freedom of choice – when, in private banks, consent is given under pressure and under the weight of the uncertainty that surrounds the possibility of suffering some disease in a distant future? When the person has been made false promises about her health? It is evident that such forms of coercion do not destroy the capacity to choose freely, but they do weaken its rational foundation and the effective range of its operation. And this is precisely what puts at risk the acceptability of the informed consent to store blood that is provided to private banks. This was evident during the early history of the public UCB of the "La Raza" National Medical Center of the Mexican Social Security Institute. It began its operation by acquiring umbilical cord samples in the "Santa Helena" private hospital. The strategy for acquiring the samples consisted in the promotion of the donation of the umbilical cord to the bank of the private hospital and for the public bank, simultaneously. Parents were informed of the advantages and disadvantages of both banks, without creating any false expectation about the possible use of the tissue. It was observed that when there is no coercion of any type parents prefer altruistic donation [Novello-Garza B.; Unpublished observations].

One corollary of autonomy is the principle of Respect for Persons. And Respect for persons requires that confidentiality and honesty (not to deceive or lie) be kept. In public banks the acquisition of samples is governed by the principles of altruism, autonomy, respect for person and confidentiality. Since private banks do not assure confidentiality and put at risk the reason for storing the umbilical cord – autologous use – they face a significant dilemma when confronted with these principles.

### Justice

Using the Mexican Constitution, which establishes that every Mexican citizen has a right to the protection of his/her health through access to the national and state health systems that guarantee it [Article 4] [45], it is logical to ask the following: Is it a duty of the Mexican government to encourage the establishment of the UCBs, and to analyze the advantages and disadvantages of public and private banks in order to determine the type of UCB that could provide the maximum benefit to the majority of the population, including its most disadvantaged sectors? Public banks through a bioarchive offer the availability of HLA compatibles for allogenic transplants, and have the possibility of extending their operation to benefit the many ethnicities that weave the fabric of Mexican society, because they are embedded in national health care systems, and networks that sometimes are international in nature. Samples stored in private banks, as has already been mentioned, can only be used for the donor and with the caveats that have been identified by scientific research [6,15,17-20]. But, what can be said of the mercantile attitude of private banks (in relation to the storage of biological materials) when this has no scientific justification? And, since these private banks serve a population well resourced and with a range of options that people with less resources have, from a moral perspective it seems that they should never be subsidized by the government, be it federal, state or local. Clinical Ethics Committees (CEC) play here a fundamental role by creating the instances in which reflection and deliberation may occur about the services, the way they are funded, and consequences of the operation of the UCBs. Policy and guideline development is seen as an important function by many CEC [46].

The notion of justice also is called into question when the distribution of the operating Mexican banks is taken into consideration. Public UCBs are located in large metropolitan areas: Mexico City (n = 2) and Nuevo León (n = 1); hence, they are not accessible to most of the Mexican population. The only bank that has attempted to be accessible and serve a more inclusive population is the Central Blood Bank at the "La Raza" National Centre. This centre is projecting to work with teams of health professionals so that they turn out at specific geographical areas in order to constitute a more complete bioarchive. It is also important to mention that umbilical cord units are granted to any patient by the public transplant centres which are located in Mexico City, Monterrey, Puebla and Guadalajara.

## Conclusion

To reflect on the UCBs' work within the field of public health policy and ethics is an opportunity to show the need for creating, developing, and modifying all those health policies that aim at reconciling goals such as informed consent, equal access to health care, health promotion and social efficiency. Inequalities in access to the banks and health care, combined with high cost of tissue storage and high recovery fees of CB units granted for transplant, give rise debates about basic medical ethics and social justice in many countries, including Mexico.

## Summary

The storage of blood from the UCBs in order to harvest hematopoietic stem cells to deal with genetic, congenital and acquired diseases appeared about 30 years ago and is coming into its own. In Mexico there are public and privately funded UCBs. However, present Mexican law is general and lacks the specificity necessary for the regulation of the UCBs. Although there are international recommendations for the control of these banks, there are certain legal and ethical aspects which are omitted from consideration. Ethical analysis allows for the evaluation of the Mexican legislation and finds the need for specific legislation to regulate the UCBs. This article is important because along with establishing the need that the Mexican State regulate and supervise, in a strict manner, the operation of both types of UCB, it shows that it is of extreme importance to bring up to date the General Health Law and the Mexican Official Norm (Ley General de Salud y la Norma Oficial Mexicana) for the appropriate use of human blood and blood derivatives in medical treatments and clinical research. The new legislation and the updating of the legislation already in place will forestall the continuation all inappropriate use of haematopoietic stem cell; and will also regulate the advertising campaigns and marketing of private banks. The new legislation should call for periodic audits of the UCBs in order to assess and very the quality of their operation.

## List of abbreviations and acronyms

CB: Cord Blood Units; CEC: Clinical Ethics Committees; CordMx: Cord blood bank in Mexico; HLA: Human Leukocyte Antigen; Netcord: International network of public cord blood banks; NOM-003-SSA2-1993: The official Mexican norm for blood transfusion; UCB: Umbilical Cord Bank; WMDA: World Marrow Donor Association.

## Competing interests

The authors declare that they have no competing interests.

## Authors' contributions

MS designed, conducted and wrote the manuscript.

BN participated in the writing-up of the manuscript.

EV participated in the conduction and writing-up of the manuscript.

All authors read and approved the final manuscript.

## Pre-publication history

The pre-publication history for this paper can be accessed here:

http://www.biomedcentral.com/1472-6939/10/12/prepub
